# Estimating the burden of acute gastrointestinal illness in the community in Gansu Province, northwest China, 2012–2013

**DOI:** 10.1186/1471-2458-14-787

**Published:** 2014-08-03

**Authors:** Xiang-Lai Sang, Xiao-Cheng Liang, Yan Chen, Jian-Dong Li, Jing-Guang Li, Li Bai, Jian-Yun Sun

**Affiliations:** Institute of Food Safety, Gansu Provincial Center for Disease Control and Prevention, No. 230 Dong Gang West Road, Chengguan District, Lanzhou, 730030 Gansu Province China; Key Laboratory of Food Safety Risk Assessment of Ministry of Health, China National Center for Food Safety Risk Assessment, No. 7 Panjiayuan Nanli, Chaoyang District, Beijing 100021 China; Department of Cerebral Surgery, Armed Police Hospital of Gansu Province, No. 253 Gong Jia Wan Road, Qilihe District, Lanzhou 730050 Gansu Province China

## Abstract

**Background:**

Acute gastrointestinal illness (AGI) imposes considerable social and economic burden on low and middle-income countries. This study aimed to estimate the occurrence, distribution, and burden of self-reported AGI in Gansu Province of northwest China, where economic growth rates have lagged far behind those of other regions in China and systematic studies to investigate the burden of AGI are still lacking.

**Methods:**

Twelve-month, retrospective face-to-face surveys were conducted in three sentinel sites between June 2012 and May 2013. Respondents were asked if they had experienced diarrhoea or vomiting in the 28 days prior to the interview.

**Results:**

In total, 2094 interviews were completed. The adjusted monthly prevalence was 8.5% with an incidence rate of 1.16 episodes of AGI per person-year. Healthcare was sought by 73.8% of those reporting illness. Of the cases who visited a doctor, 50.5% submitted a stool sample. The use of antibiotics was reported by 65.6% of the cases and 53.3% took antidiarrhoeals. In the multivariable model, age, household income and sentinel site were significant risk factors of being a case of AGI.

**Conclusions:**

The burden of AGI was considerable in Gansu Province of northwest China. Ongoing research to identify the main causes of AGI is needed for more accurate estimate of the burden of AGI in this region.

**Electronic supplementary material:**

The online version of this article (doi:10.1186/1471-2458-14-787) contains supplementary material, which is available to authorized users.

## Background

Acute gastrointestinal illness (AGI) is a common illness all over the world. The aetiological factors of AGI include viruses, bacteria, parasites, toxins, and metals. The symptoms of AGI typically include diarrhoea and/or vomiting. In low and middle-income countries, this disease is one of the leading causes of morbidity and mortality, especially in children [1]. Even in industrialized countries, the social and economic burden caused by AGI were reported to be substantial due to its high morbidity, although its mortality is low in these countries [2–6]. The social and economic burden of AGI would be considered to be significant in China.

To estimate the burden of AGI to the society in China, the China National Center for Food Safety Risk Assessment (previously known as the Food Safety Section of the National Institute for Nutrition and Food Safety, Chinese Center for Disease Control and Prevention) conducted a population-based study to describe community situations of AGI in several provinces during 2010–2011 [7, 8]. However, systematic studies to investigate the burden of AGI in northwest China were still lacking. In this region, economic growth rates have lagged far behind those of other regions in China and their basic medical services need to be improved. Therefore, estimation of the burden caused by AGI within the population is of particular importance for better utilization of the limited medical services, and ultimately, for establishment of preventive strategies.

Reports from a number of countries suggest that rates of AGI in a community depend on person, place and time [9, 10]. In Gansu Province, as there are distinct regional differences in climate and environmental conditions, variable in AGI rates and situations, including social burden, were expected across the Province. Therefore, the objectives of the current study were to estimate the occurrence, distribution, and burden of self-reported AGI in Gansu Province located in northwest China.

## Methods

### Study design

**Figure 1:**
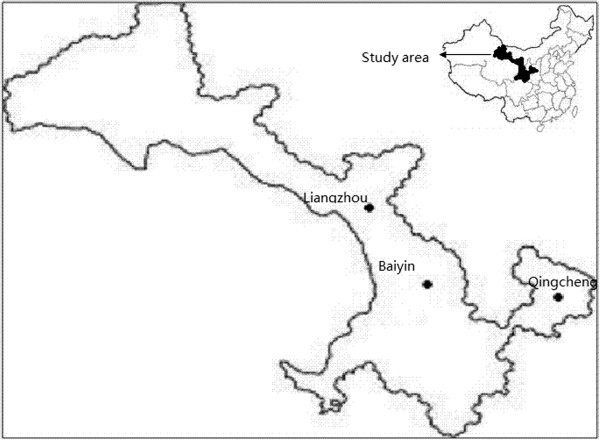
**Map of the sentinel sites in Gansu Province, northwest China.**

Twelve-month, cross-sectional, face-to-face surveys were conducted between June 2012 and May 2013 within three purposefully-selected sentinel sites in Gansu Province (Figure 1). The sentinel sites were selected based on their geographic location, population density, level of economic development and predefined risk rating. The three sentinel sites selected were: (a) Baiyin District (1372 sq km, population–294400) in Baiyin Prefecture, (b) Liangzhou District (5081 sq km, population–1010295) in Wuwei Prefecture, and (c) Qingcheng County (2673 sq km, population–261898) in Qingyang Prefecture. The sentinel sites represent approximately 6.1% of the total permanent resident population (25575263) in 2010 in Gansu Province. Each sentinel site was divided into several blocks in accordance with the administrative division, resulting in a total of 71 blocks in the study. From each block, a city block in urban areas or a village in rural areas was randomly selected. Households were randomly selected from each city block or village using a random number table in such a way as to make the number of households in the survey proportional to its population size.

A target sample size of 2046 (i.e. 2046 interviews) was calculated to detect a prevalence of 10%, with 1.3% allowable error and 95% confidence (Epi Info v. 3.5.3, CDC, USA). The number of respondents varied among the sentinel sites since the sample size was determined to be proportionate to the population size of the sentinel site. However, it was constant among months within the same site.

Within each household, the person who was next to celebrate a birthday was selected to participate in the survey. If the selected person declined or no one lived at the residence, the neighbouring household was selected as a replacement. Proxy respondents were used for study participants aged < 12 years and for individuals aged between 12 and 16 years when parental or guardian consent was not given to interview them directly. Written and informed consent was sought from all respondents before the interview. All interviews were administered in Chinese. The study was approved by the ethic committee of Gansu Provincial Center for Disease Control and Prevention.

### Interviews

Trained health workers employed in rural hospitals and community health service organizations conducted face-to-face interviews. Data were collected using a questionnaire developed through the modification of a pre-existing population-based burden-of-disease questionnaire [8]. For each participant, in addition to general demographic data, information was collected on the occurrence of diarrhoea or vomiting during the 4-week before the interview. Upon positive answers, participants were asked about background illness and medication, including chronic gastrointestinal illness, pregnancy and other potential competing causes of gastrointestinal symptoms such as usage of drugs and alcohol. Furthermore, they were also asked about the details of symptoms and timing, perceived causes of their illness, medical consultations and treatment, admission to hospital, whether a stool sample was sent for diagnostic purposes, the social and economic impact of illness, and the occurrence of AGI in other household members. Additional file 1 is the translated questionnaire used in the survey.

### Case definition

A case of gastroenteritis was defined for this study as an individual with ≥ 3 loose stools, or any vomiting, for a period of 24 h during the 4 weeks prior to the interview, but excluding those (a) with colorectal cancer, Crohn’s disease, irritable bowel syndrome, colitis, diverticulitis of large intestine, or another chronic illness with symptoms of diarrhoea or vomiting diagnosed by a doctor, or (b) who report their symptoms were due to non-infectious causes such as pregnancy, excess alcohol, chemotherapy/radiotherapy, drugs, menstruation, or food allergy. This definition is compatible with the case definition proposed by the International Collaboration on Enteric Disease ‘Burden of Illness’ Studies [11]. Respondents who identified more than one episode of AGI, separated by seven days or more, during the 4 weeks prior to the interview were asked to report only their most recent episode.

### Statistical analysis

Data were entered into EpiData version 3.1 (EpiData Association, Odense M, Denmark) creating one database for each sentinel site. All the data analyses were performed using the SPSS version 16.0 (SPSS Inc., Chicago, IL, USA). Yearly household income was classified as below 20000 China Yuan (CNY) (equivalent to ca. 2800 US Dollar (USD)), 20000–50000 CNY (2800–7000 USD) and above 50000 CNY (7000 USD). A variable named ‘season’ was created based on the date of interview, as already used in a previous study [8]: winter (January to March), spring (April to June), summer (July to September) and fall (October to December).

The response rate was calculated by dividing the number of completed surveys by the number of households visited. The monthly prevalence of AGI was calculated by dividing the number of respondents reporting AGI in the 4 weeks prior to the interview by the total number of respondents. The point prevalence of AGI was obtained as the proportion of cases with symptoms on the day of the interview. The proportion of seeking medical care for AGI was calculated by dividing the number of respondents who saw a doctor for his/her disease by the number of AGI cases. The proportion of stool sample submission was the proportion of cases whose stool samples were taken by doctors for laboratory testing to the total AGI cases. For incidence rates, even a respondent reported multiple episodes during the 4-week period prior to the interview, the case was counted in a ‘single episode’. The Incidence rate was calculated using the formulae obtained from the book, Modern Epidemiology [12].

These estimates were adjusted for known differences between the survey sample and the target population by weighting for age, sex and residence using the Sixth National Population Census data of Gansu Province as the reference population [13]. The association between demographic factors and the occurrence of AGI was tested using the χ2 test. The mean duration of illness in different age groups was compared by analysis of variance (ANOVA). The multivariable logistic regression model was used to estimate odds ratios (ORs) for each of the geographic and demographic variables. The goodness-of-fit of the model was assessed with the Hosmer–Lemeshow statistic, with an adequate calibration indicated by p > 0.05. In multivariable analysis, forward selection method was used to select variables based on the Wald χ2 test until all variables remaining in a model were significant at p < 0.05. Explanatory variables tested were age, education, household income, household size, household type, residence, season, and sentinel site.

## Results

### Response rate and representativeness of the sample

The response rate was 86.0% with 2094 valid questionnaires. In general, survey respondents were older, residing in larger households, and more likely to be males. Similarly, respondents residing in rural areas were overrepresented (Table 1).

**Table 1:** **Demographic characteristics of respondents and weighted monthly prevalence of acute gastrointestinal illness (AGI) in the 4 weeks prior to interview in Gansu Province, northwest China, June 2012**–**May 2013 (n = 2094)**

Variable	Proportion of residents	Proportion of survey respondents	Monthly prevalence of AGI		P-value
			%	(95% CI)	
Sex					0.859
Male	51.1	75.5	8.6	(6.9–10.3)	
Female	48.9	24.5	8.4	(6.7–10.1)	
Age (years)					< 0.01
0–4	5.4	2.1	12.4	(6.3–18.5)	
5–14	12.7	3.2	19.2	(14.4–23.9)	
15–24	18.6	4.3	11.9	(8.7–15.2)	
25–44	32.6	33.5	5.2	(3.5–6.9)	
45–64	22.4	43.6	4.4	(2.6–6.3)	
≥ 65	8.2	13.3	5.9	(2.4–9.4)	
Education					< 0.01
Preschool children	6.5	2.4	11.5	(6.2–16.8)	
Illiterate	9.6	10.0	7.3	(3.4–11.2)	
Primary school	32.5	29.3	11.4	(8.7–14.1)	
Secondary school	31.2	40.6	4.7	(3.2– 6.2)	
High school and above	20.2	17.6	10.5	(7.8–13.2)	
No response^a^		— ^b^			
Yearly household income (CNY)					< 0.01
0–19999	NA	26.0	3.3	(1.6–5.0)	
20000–49999	NA	63.7	9.1	(7.6–10.6)	
≥ 50000	NA	9.6	13.7	(9.7–17.6)	
No response^a^		0.7			
Household size (number of person)					< 0.01
1–2	29.9	18.4	3.1	(1.1–5.0)	
≥ 3	70.1	81.6	9.4	(8.1–10.8)	
Household type					0.013
No residents < 18 years	NA	42.6	6.5	(4.7–8.2)	
At least one resident < 18 years	NA	57.4	9.7	(8.1–11.3)	
Residence					< 0.01
Urban	35.9	23.5	11.7	(9.4–14.0)	
Rural	64.1	76.5	6.7	(5.4–8.1)	
Season					0.045
Winter	NA	25.0	8.1	(5.9–10.3)	
Spring	NA	25.2	9.7	(7.1–12.2)	
Summer	NA	24.7	10.8	(7.8–13.7)	
Autumn	NA	25.1	6.0	(4.0–8.0)	
Sentinel site					< 0.01
Baiyin	18.8	16.5	1.4	(0.2–2.6)	
Liangzhou	64.5	62.8	5.6	(4.4–6.9)	
Qingcheng	16.7	20.7	24.0	(19.9–28.1)	

CI, Confidence interval; NA, Not available; CNY, China Yuan.

^a^Individuals who did not respond were excluded from analysis.

^b^Value is less than 0.1.

### Frequency and distribution of AGI

Of the 2094 respondents in the survey, 110 (5.3%) reported having symptoms of gastroenteritis in the previous 4 weeks. Of these respondents, six were identified as non-infectious causes and included in the non-case category, leaving 104 respondents to be identified as cases. After excluding these respondents, an overall prevalence of AGI in the 28 days prior to the interview, adjusted for age, sex, and residence, was 8.5% (95% Confidence Interval (CI) 7.3–9.7). This represents an average of 1.16 (95% CI 1.14–1.18) occurrences of AGI per person-year. A total of nine people still had symptoms of AGI at the time of interview, yielding an unadjusted point prevalence of 0.4% (95% CI 0.2–0.7) and age, sex, and residence adjusted prevalence of 1.8% (95% CI 1.2–2.3). Of AGI cases, 9.6% (10/104) also reported respiratory symptoms which included nasal congestion, sneezing, runny nose, coughing, sputum sore throat and otitis.

**Figure 2:**
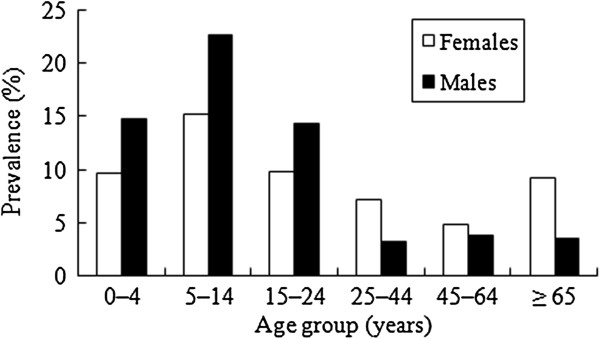
**Monthly prevalence of acute gastrointestinal illness, by age and sex, in the 4 weeks prior to interview in Gansu Province, northwest China, June 2012–May 2013.**

**Figure 3:**
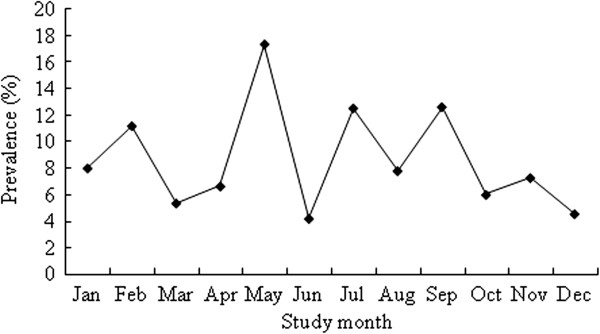
**Monthly prevalence of acute gastrointestinal illness, by month, in the 4 weeks prior to interview in Gansu Province, northwest China, June 2012–May 2013.**

Estimates of the monthly prevalence of AGI in population with specific demographic characteristics are presented in Table 1. There was no significant difference in the prevalence of AGI between males and females (8.6% *vs* 8.4%). Children aged 0–4 (12.4%) and 5–14 (19.2%) had higher prevalence of AGI. For age groups 0–4, 5–14 and 15–24, the monthly prevalence of AGI was higher in males than females, and for the rest of age groups, the opposite tendency was observed (Figure 2). Of all the respondents, 81.6% households had ≥ 3 members in the household. The prevalence of AGI was significantly higher in these larger households (9.4%) than in those with 1–2 members in a household (3.1%) (p < 0.01). Respondents living in a household with at least one person aged < 18 years were more likely to have experienced AGI than those without (p < 0.05). The prevalence of AGI was the highest in the population living in households with incomes over 50000 CNY and declined with the decrease of household income. There was a difference in the prevalence of AGI between the urban (11.7%) and rural areas (6.7%) (p < 0.01). The AGI prevalence was significantly associated with season, with the highest prevalence in summer (p < 0.05). However, while the average rates are higher during summer, the actual peak of illness occurred in spring (Figure 3). This lack of consistent trend by season coincides with the variable ‘season’ being excluded from the multivariable model. Prevalence varied by sentinel site, being highest in Qingcheng (24.0%), followed by Liangzhou (5.6%) and Baiyin (1.4%), with statistical significance (p < 0.01).

### Multivariable analysis

The final multivariable model includes age, household income and sentinel site (Table 2). Children aged 5–14 years were more likely to report AGI. Compared with the lowest income groups, the higher income groups had increased odds of reporting AGI. Respondents who lived in Qingcheng were more likely than in other sites to report AGI.

**Table 2:** **Multivariable model of risk factors associated with acute gastrointestinal illness in the 4 weeks prior to interview in Gansu Province, northwest China, June 2012**–**May 2013**

Variable	OR	95% CI	P-value
Age (years)			< 0.01
0–4	1.89	0.75–4.80	0.18
5–14	3.35	1.56–7.21	< 0.01
15–24	2.25	0.98–5.14	0.06
25–44	0.87	0.52–1.44	0.58
45–64	Ref.	Ref.	Ref.
≥ 65	1.17	0.58–2.36	0.66
Yearly household income (CNY)			< 0.01
0–19999	Ref.	Ref.	Ref.
20000–49999	2.34	1.29–4.25	< 0.01
≥ 50000	4.05	1.89–8.69	< 0.01
Sentinel site			< 0.01
Baiyin	0.10	0.04–0.25	< 0.01
Liangzhou	0.23	0.15–0.35	< 0.01
Qingcheng	Ref.	Ref.	Ref.

OR, Odds ratio; CI, Confidence interval; CNY, China Yuan.

Hosmer and Lemeshow test (p = 0.156).

### Symptoms and severity

Among the 104 cases, 74 (71.2%) had diarrhoea only, 3 (2.9%) had vomiting only, while the remaining 27 (26.0%) had both of them. Abdominal cramps (84.6%), loss of appetite (52.9%) and nausea (37.5%) were also reported at high rates. Of 101 (i.e., 74 + 27) respondents with diarrhoea, 1 (1.0%) had bloody diarrhoea. Of the 104 cases with AGI, 7 (6.7%) reported more than one episode in the previous 28 days.

Among the 95 cases whose illness had resolved at the time of interview, the mean duration of illness was 2.5 days (range 0.3–25 days) and the median was 2.0 days. There was no significant difference in the duration of illness among different age groups (p > 0.05, ANOVA). On the worst day of symptoms, cases reported an average of 4.7 times (range 3–10 times) loose stools and 2.7 times (range 1–6 times) vomiting.

### Suspected cause of illness

Of those AGI cases who responded to a question about potential cause of their illness, 35.9% (37/103) did not suggest a possible cause for their illness. Those suggesting what they believed was the cause thought their illness was due to consuming contaminated food (86.4%) or water (1.5%), or other causes (12.1%). Of the 57 cases who suspected that their illness was due to eating contaminated food, 40.4% thought it was due to fruits and vegetables, 31.6% due to meat and meat products, 8.8% due to cereals and cereal products. Of these 57 cases, 70.2% thought that the contaminated food was what was served at home, and 19.3% from street vendors.

### Healthcare seeking behaviour

Healthcare-seeking behaviour, medication use and the source of the medication are shown in Table 3. In total, 75.0% of the cases visited a doctor. After adjusting for age, sex and residence, the prevalence of seeing a doctor was 73.8% (95% CI 67.3–80.4). County level hospitals were the medical institutes sought most frequently (56.4%), followed by rural hospitals (33.3%) and clinics (7.7%). Among those who saw a doctor, 48.7% reported having provided a stool sample for laboratory testing. After adjusting for age, sex and residence the proportion of stool sample submission among those who saw a doctor was 50.5% (95% CI 41.8–59.2). ‘Self-medication’ (76.0%) and ‘not thinking the illness was severe enough to seek medical care’ (72.0%) were the most common reasons for not seeing a doctor. Twenty one percent (22/104) of AGI cases hospitalized for at least 1 night. Of the 11 cases who reported the duration of hospitalization, the mean duration was 3.6 night (range 2–5 nights), and the median was three nights.

**Table 3:** **Hospital visit and medicine use by cases of acute gastrointestinal illness in the 4 weeks prior to interview in Gansu Province, northwest China, June 2012**–**May 2013**

Variable	No. of case (%)
Sought medical care (n = 104)	
Yes	78 (75.0)
No	26 (25.0)
Submit a stool sample (n = 78)	
Yes	38 (48.7)
No	40 (51.3)
Take medicine (n = 104)	
Yes	98 (94.2)
No	6 (5.8)
Type of medicine (n = 92)^a^	
Antibiotics	64 (69.6)
Antidiarrhoeals	52 (56.5)
Analgesics	19 (20.7)
Antipyretics	9 (9.8)
Antacids	3 (3.3)
Other	5 (5.4)
Unknown	19 (20.7)
Location of medicine purchase (n = 98)^a^	
Hospitals with prescription	78 (79.6)
Family medicine chest	16 (16.3 )
Pharmacy	12 (12.2 )
Other	1 ( 1.0 )

^a^Because some cases took more than one type of medication and some cases visited more than one location, so the total percentage may exceeds 100%.

In total, 94.2% cases took medications to treat or relieve symptoms for their illness. About seventy percent (65.6%) of those with AGI were estimated to have taken antibiotics, while 53.3% reported taking antidiarrhoeals. Of those who took medicine, 79.6% reported that the medication was prescribed by a doctor, 16.3% used medicine in the family medicine chest, and 12.2% bought medicine from a pharmacy without prescription.

### Impact on work and school

The illness forced three (3.8%) of the 80 employed or self-employed patients to miss work. The mean number of days taken off work was 2.3 days (range 1–5 days). The illness forced four (33.3%) of the 12 student patients off school. The mean number of days of school absenteeism was 1.8 days (range 1–4 days). Of persons with AGI, 1.9% (2/104) reported that at least one another person living in the same household had suffered from AGI during the 28 days prior to the interview.

### Standard case definition comparison

The proposed minimum set of results of this study, thus allowing for international comparisons are outlined in Table 4.

**Table 4:** **Epidemiology of acute gastrointestinal illness** ^**a**^ **under the standard case definition**

Incidence per person-year (95% CI)	1.16 (1.14–1.18)
Incidence per person-year in males	1.17
Incidence per person-year in females	1.14
Mean age of cases (years)	39.49
Mean duration of illness (days)	2.48
Cases with bloody diarrhoea (%)	0.96
Cases who sought medical care (%)	73.84
Cases submitting a stool sample for testing (%)	37.28
Cases with respiratory symptoms (%)	9.60
Cases with symptoms still ongoing at time of at interview (%)	8.65

CI, Confidence interval.

^a^Acute gastrointestinal illness was defined as diarrhoea of ≥ 3 loose stools or any vomiting in a 24-hour period.

## Discussion

This study provides the first population-based estimates of the occurrence, distribution, and burden of AGI in Gansu Province of northwest China. The results indicate that AGI leads to a substantial burden in this province. Based on the estimates of 1.16 AGI episodes per person-year and 73.8% of cases seeking medical care for AGI in this study, 29.6 million cases of AGI and 21.9 million medical consultations are estimated to occur each year in Gansu Province. The mean duration of illness was 2.5 days. If extrapolated to the population, 73.4 million days of illness due to AGI occur in the Gansu population each year.

We compared our results with the recently performed retrospective studies in other developing countries using compatible case definitions. The monthly prevalence of 8.5% in this study is higher than those reported for Jamaica (4.0%), Malaysia (5.0%), but lower than those reported for Chile (9.2%) and Cuba (10.6%) [9, 14–16]. The AGI prevalence estimated in the Gansu population is also higher than those reported for other regions of China [8, 17]. In the northwest parts of China, like many other developing regions in the world, people’s living standard is yet to be improved. Substandard hygiene and sewage disposal, inadequate facilities for food storage and preparation, and lack of appropriate drinking water are factors that may contribute to the high incidence rate of AGI in this region.

The methods for case ascertainment significantly impact incidence estimates. A UK study shows that the retrospective AGI study estimated 2–3 times higher incidence in the community than the prospective study [18]. This discrepancy was once attributed to telescoping, a recall bias leading respondents to attribute to the survey period episodes of AGI that occurred earlier. However, an evidence also exists that the shorter 7-day recall period yields significantly greater annual estimates compared to the longer 30-day recall period, which is contrary to telescoping resulting in the true burden of disease being underestimated [16, 19, 20]. Standardizing these methodological choices is important if valid estimates of the burden of AGI from different countries are sought.

Because it is not mandatory to report sporadic foodborne illnesses to a specific agency in China, the number on foodborne infection and intoxication cases do not accurately reflect, but tends to be lower than real cases. In 2012, only 397 foodborne disease cases were reported to the Gansu Provincial Foodborne Diseases Surveillance Network (data not shown). The total number of AGI in a study multiplied by the proportion of foodborne AGI gives the total number of cases of foodborne AGI in the region. Many of the foodborne AGI estimates in the developed countries are established using cause-specific estimates [21–23]. Without any pathogen attribution, such extrapolation is erroneous. Further research into the pathogen-specific burden of AGI is necessary to better estimate the burden of AGI and foodborne disease in this region.

When the cases of AGI with concurrent respiratory symptoms were excluded from the analysis, the incidence rate dropped to 1.04 AGI episodes per person-year, a reduction of 10.5%. This variation falls within the range of those reported by similar surveys conducted in the USA, Canada, Australia, and Italy [24, 25]. As some AGI cases with respiratory symptoms may be due to respiratory infections, it is necessary to consider this issue when estimating the burden of foodborne disease based on data from population studies.

The highest rate of disease in children aged 5–14 years is unique, as previous studies suggest highest rates of AGI among children aged 0–4 years [14, 16]. In the present study, the AGI prevalence was higher for young male groups. Young adults and males are more likely to engage in such food handling leading to higher risk than others, which may be due to the lack of food safety education/training, role models, and/or food-handling experience [26]. Additionally, this study found a higher rate of AGI in females aged >25 years, as in other studies [8, 27]. Having far higher occasions in handling food and caring for children may induce females more frequently exposed to enteric pathogens than males. Identification of groups of people vulnerable to AGI is useful to prioritize resource allocation. Food safety education should be started during childhood and should reach out to the mass of people.

There is a strong link between climate-related events, farming activities and the occurrence of AGI [10, 20, 28, 29]. Gansu Province is predominantly an agricultural area, which has complex landforms and multiple climate types. Baiyin is an industrial district and there was lower risk of AGI, which may be attributed to the absence of livestock and, consequently, their drinking water sources being less contaminated by agricultural run-off. The economic level is higher in Baiyin than in the other two sites, and the prevalence of AGI in Baiyin is close to those in some developed regions of China, such as Shanghai [8]. Liangzhou is located at the edge of dessert, and land desertification has caused many agricultural populations migrate to cities or other places, or to make a living in other means. The lower risk of AGI might be related to the reduced rainfall and the reduced farming activities. Qingcheng showed a significantly higher risk than in the other sites. The weather in Qingcheng is warm and humid, which is appropriate for intensive farming activities.

The proportion of cases reporting that consuming contaminated food (55.5%, 57/103) was a cause of their illness was higher than previous studies [8, 17, 30]. In fact, people seldom really know the cause of their AGI, although they usually attribute it to food. However, if there is no better way to estimate the proportion of foodborne AGI, this subjectively obtained proportion may be used to roughly estimate the burden of foodborne disease [8, 31]. Further research into the pathogen-specific burden of AGI is needed to estimate the foodborne proportion of AGI more accurately.

The proportions of cases who visited a doctor (73.8%) and who were hospitalized (21.2%) found in this study were higher than the proportions observed in other regions of China and in those countries cited above [8, 11, 17, 25, 32, 33]. These differences may reflect important differences in healthcare systems in the studied countries. However, the fact that a relatively high proportion of cases visited a doctor and hospitalized may also suggest that this study is primarily capturing the severe cases.

Of the respondents with AGI who visited a doctor, 50.5% provided a stool sample, which was higher than in other countries [11, 25, 32, 33]. If there are 21.9 million medical consultations each year in Gansu Province, the number of stool exam is 11.0 million. This provides a rich opportunity to document pathogen-specific illness rates. However, when a stool specimen is received, it is routinely tested for white blood cells but not cultured for pathogenic bacteria. Although clinical laboratories often performed tests of unproven value, the fact that the practice of seeking health care and submitting a stool sample is important. The proportion of seeking medical care for AGI and the proportion of stool sample submission will help to estimate the size of each layer in the surveillance pyramid for AGI.

Antibiotics are very rarely prescribed for the treatment of AGI. However, their use was reported by 65.6% of those with AGI, much higher than in other countries [9, 16, 25, 32, 34, 35]. However, proportion of antibiotic use in Gansu population was similar to those reported by other regions of China, except Hong Kong [8, 17]. If there are 29.6 million episodes of AGI each year in Gansu province, the number of AGI cases who are treated with antibiotics may be 19.4 million. This is of particular concern, particularly in view of the increase in antibiotic resistant pathogens and human susceptibility to ingested pathogens.

There was a tendency for Gansu population with higher household incomes to be more likely to report AGI, quite different from findings in Malaysia and Malta [15, 36], but similar to those in Australia and the United States [10, 37]. In a previous study carried out in other regions of China, the multivariate analysis showed that the variation across income groups was not significant [8]. This is possibly due to a reporting bias, with more wealthy households more likely to perceive that they have symptoms, pay more attention to hygienic conditions, or may be due to such behaviours in this group as eating out and travelling more frequently, leading them greater risk of acquiring AGI. Further research is needed to understand the relationship between household incomes and the occurrence of AGI in this region.

The response rate was quite high in this study compared to surveys in those countries cited above. This high rate may be attributed to: (1) a face-to-face interview employed to enable us to reach out to potential participants who do not have access to a telephone; and (2) the surveys carried out by trained health workers whom most people in Gansu Province regard highly.

## Conclusions

We conclude that acute gastrointestinal illness (AGI) represents a substantial burden on health and economy in Gansu Province in northwest China. Ongoing research to identify the main causes of AGI is needed for more accurate estimate of the burden of AGI in this region.

## Electronic supplementary material

Additional file 1: **Study questionnaire.** (DOC 60 KB)

## Abbreviations

AGI: Acute gastrointestinal illness
ANOVA: Analysis of variance
CI: Confidence interval
NA: Not available
OR: Odds ratio.
