# Supplementary material for: Estimating the burden of acute gastrointestinal illness in the community in Gansu Province, northwest China, 2012–2013
Source: BMC Public Health. 2014 Aug 3;14:787. doi: 10.1186/1471-2458-14-787 (PMC4246514; doi:10.1186/1471-2458-14-787)
Supplement: Supplementary file 1 — Additional file 1: Study questionnaire. (DOC 60 KB) [file 12889_2013_7281_MOESM1_ESM.doc]

**Study Questionnaire**

T1. Date of interview Years/Months/Days

**Section one: Basic information**

N1. Sentinel site code _______________

N2. Person code ________________

N3. If the questionnaire being answered by the selected respondent him or herself?

[1] Yes [2] No

N4. How many people are usually live in this household? ______

N4a. How many are <18 years of age? ______(enter ‘0’ if none)

N5. Total family income per year ___________ yuan

N6. Home address

Province ________ Prefecture ________ District/County ____________

Township/Street ___________ Village/Community ______________

N7. Residence [1] Urban [2] Rural

N8. Telephone ____________

N9. What is your (your child’s) name? _________________

N10. Gender [1] Male [2] Female

N11. Age ___ years

N12. Ethnic group [1] Han [2] Manchu [3] Mongol [4] Hui [5] Tibetan [6] Miao

[7] Zhuang [8] Uyghur [9] Other

N13. Education

[1] Preschool children [2] Illiterate [3] Primary school [4] Secondary school

[5] High school [6] Technical secondary school and junior college

[7] University [8] Postgraduate

N14. Occupation

[1] Child in child care settings [2] Child outside child care settings [3] Student

[4] Teacher [5] Childcare worker and housekeeper

[6] Employed persons in catering services [7] Service personnel in public places

[8] Business service personnel [9] Medical personnel [16] Worker

[17] Migrant labourer [18] Farmer [19] Herdsman [20] Fisherman

[21] Sailor and long-distance driver [22] Administrator and office staff [23] Retired

[24] Home and unemployed [28] Unknown [29] Other

**The past 2 weeks, that is from (date) through (date) .**

N15. During the past two weeks did you (your child) travel outside of the district/county where your household resides? [1] Yes [2] No (go to N16)

N15.1. To which place did you (your child) travel? ___________

**Section two: Symptoms**

**The past 4 weeks, that is from (date) through (date) .**

N16. During the past 4 weeks, have you (your child) suffered from diarrhea or vomiting? Diarrhoea was ≥ 3 loose stools in a 24-hour period.

[1] Yes [2] No (go to N18)

N17. Whether you were diagnosed by a doctor (a) with colorectal cancer, Crohn’s disease, irritable bowel syndrome, colitis, diverticulitis of large intestine, or another chronic illness with symptoms of diarrhoea or vomiting, or (b) who report their symptoms were due to non-infectious causes such as pregnancy, excess alcohol, chemotherapy/radiotherapy, drugs, menstruation, or food allergy? [1] Yes [2] No

N17.1. If yes, which illness? _____________________

N18. Judged by the interviewer, how many episodes of acute gastrointestinal illness did the respondent have during the past 4 weeks? ____ [(1) enter ‘0’ if N16 = 2; (2) enter ‘0’ if n17=1; (3) a 7-day symptom-free interval was defined to distinguish multiple episodes]

**If N18 = 0, go to the place of signature.**

If N18 ≥ 1, respondents were asked to respond only for their most recent episode for the remaining questions.

N19. Have you (your child) suffered from loose stools? [1]Yes [2] No (go to N20)

N19.1. If yes, how many times in a 24 hour period? ____

N19.2. Have you (your child) suffered from bloody diarrhoea? [1]Yes [2] No

N19.2.1. If yes, how much blood was there in your (your child’s) stool? ___

[1] Just a little blood on the toilet paper

[2] Some blood mixed with the stool

[3] So much blood that the stool was almost entirely blood

N20. Have you (your child) suffered from vomiting? [1] Yes [2] No (go to N21.1)

N20.1 If yes, how many times in a 24 hour period? ___

N21. Have you (your child) also experience the following symptoms?

N21.1. Nausea [1] Yes [2] No

N21.2. Abdominal pain [1] Yes [2] No

N21.3. Lose of appetence [1] Yes [2] No

N21.4. Fever [1] Yes [2] No

N21.5. Headache [1] Yes [2] No

N21.6. Muscle pain [1] Yes [2] No

N21.7. Joint pain [1] Yes [2] No

N21.8. Others, please specify ______________ (symptoms here except those belong to respiratory system)

N22. Have you (your child) experience the following respiratory system symptoms?

N22.1. Nasal congestion [1] Yes [2] No (go to N23)

N22.2. Sneezing [1] Yes [2] No

N22.3. Runny nose [1] Yes [2] No

N22.4. Coughing [1] Yes [2] No

N22.5. Sputum [1] Yes [2] No

N22.6. Sore throat [1] Yes [2] No

N22.7. Otitis [1] Yes [2] No

N22.8. Others, please specify ______________

N23. Are you (your child) still suffering from symptoms of acute gastrointestinal illness today? [1] Yes (go to N25) [2] No

N24. How long did the illness last? Days _____ Hours ____

N25. In your opinion, what do you think was the most possible cause of your (your child’s) illness?

[1] Food poisoning [2] Person-to-person [3] Contaminated water

[4] Animal contact [5] Other [6] Unknown

N25.1 Other, please specify _______

**Section three: Suspected food**

N26. If food poisoning, which food you think was most suspected to cause your (your child’s) symptoms? ______

N26.1. Type of food

[1] Meat and meat products [2] Milk and dairy products

[3] Eggs and egg products [4] Fishery products [5] Cereals and cereal products

[6] Beans and bean products [7] Vegetable Oil [8] Fruits and vegetables

[9] Other [10] Unknown

N27. If food poisoning, where do you think you (your child) got the food that caused your (your child’s) symptoms?

[1] Own home [2] Private house (excluding own home) [3] Hotel/Restaurant

[4] Fast food service [5] Food supermarket [6] Street vendor [7] Takeaway

[8] School cafeteria [9] Company cafeteria [10] Food service on construction sites

[11] Other [12] Unknown

N27.1. Other, please specify___________________

**Section four: Medical treatment**

N28. Did you seek medical care for this illness? [1] Yes [2] No (go to N34)

N29. How many days after your illness, you have sought medical care? ____________

N30. Name of medical institute _________________

Type of hospital grade (select multiple)

N30.1. Tertiary hospital [1] Yes [2] No

N30.2. Secondary hospital [1] Yes [2] No

N30.3. Children's hospital [1] Yes [2] No

N30.4. Center of Community Health Service [1] Yes [2] No

N30.5. County hospital [1] Yes [2] No

N30.6. Rural hospital [1] Yes [2] No

N30.7. Clinic [1] Yes [2] No

N30.8. Others [1] Yes [2] No

N31. As a result of this illness whether you (your child) hospitalized? [1] Yes [2] No (go to N33)

N31.1. If yes, how many days were you (your child) hospitalized? ___

N32. Name of hospital you admitted in ___________________________

Type of hospital grade you admitted in (select multiple)

N32.1. Tertiary hospitals [1] Yes [2] No

N32.2. Secondary hospital [1] Yes [2] No

N32.3. Children's hospital [1] Yes [2] No

N32.4. Center of Community Health Service [1] Yes [2] No

N32.5. County hospital [1] Yes [2] No

N32.6. Rural hospital [1] Yes [2] No

N32.7. Clinic [1] Yes [2] No

N32.8. Others [1] Yes [2] No

N33. Were you (your child) asked to submit a stool sample? [1] Yes [2] No

N33.1. The result of the stool sample ___________ (enter the aetiology being identified by the laboratory, if not sure, enter ‘unknown’)

N34. Reason for not seeing a doctor

N34.1. Did not think it was severe enough to seek medical care [1] Yes [2] No

N34.2. Self-medication [1] Yes [2] No

N34.3. Too busy [1] Yes [2] No

N34.4. The expenditure for seeking healthcare is high [1] Yes [2] No

N34.5. Transportation problems [1] Yes [2] No

N34.6. Have no medical insurance [1] Yes [2] No

N34.7. Distrust doctors [1] Yes [2] No

N34.8. The healthcare environment is not good enough [1] Yes [2] No

N35. Did you (your child) take any medications for this illness? [1] Yes [2] No (go to N38)

N35.1. Pharmacy [1] Yes [2] No

N35.2. Hospitals with prescription [1] Yes [2] No

N35.3. Family medicine chest [1] Yes [2] No

N35.4. Other, please specify ___________________

N36. How many days were medications taken for? _________

N37. Name of the medication(s) (enter ‘unknown’ if not sure)

Type of medicine

N37.1. Antibiotics [1] Yes [2] No

N37.2. Antidiarrhoeals [1] Yes [2] No

N37.3. Analgesics [1] Yes [2] No

N37.4. Antipyretics [1] Yes [2] No

N37.5. Antacids [1] Yes [2] No

N37.6. Other [1] Yes [2] No

N37.7. Unknown [1] Yes [2] No

**Section five: Social and economic impact of illness**

N38. Cases

N38.1. Cost of medication by case who did not visit a doctor _______ yuan (enter ‘0’ if none)

N38.2. Travel cost by case who did not visit a doctor _______ yuan (enter ‘0’ if none)

N38.3. Cost for out-patient treatment _______ yuan (enter ‘0 ’if none)

N38.4. Cost for inpatient treatment _______ yuan

N38.5. Travel cost for medical care _______ yuan (enter ‘0’ if none)

N38.6. Additional cost of food and accommodation _______ yuan (enter ‘0’ if none)

N38.7. Did this illness require you (your child) to miss work or school/college?

[1] Yes [2] No (go to N39)

N38.7.1. No. of days missed from work

N38.7.2. No. of days missed from school/college

N39. Visitors

N39.1. How many times did someone visit you (your child) while you (your child) were in the hospital? _______ (enter ‘0’ if none, and go to N40)

N39.2. Travel cost _______ (enter ‘0’ if none)

N39.3. Additional cost of food and accommodation _______ yuan (enter ‘0’ if none)

N39.4. No. of days missed from work by visitors _______ (enter ‘0’ if none)

N40. Caregivers

N40.1. No. of caregivers _______ (enter ‘0’ if none, and go to N41)

N40.2. Travel cost _______ yuan (enter ‘0’ if none)

N40.3. Additional cost of food and accommodation _______ yuan (enter ‘0’ if none)

N40.4. No. of days missed from work by caregivers _______ (enter ‘0’ if none)

N40.5. Cost of paid help _______ yuan (enter ‘0’ if none)

N41. Did anyone else in your household have suffered from acute gastrointestinal illness in the past 4 weeks? If any, how many? _______ (enter ‘0’ if none)

Name of interviewer ______________ Name of assessor _____________
